# Monitoring Hygiene Protocols and Exploring Alternatives to Counteract Resistant Pathogens: A Case Study from Southern Italy on Healthcare-Associated Infection Control

**DOI:** 10.3390/microorganisms14061382

**Published:** 2026-06-22

**Authors:** Enza Mallardo, Claudio Attilio Baliano, Valeria Pedata, Rosita Zinzi, Federica Mayella, Mauro Murano, Antonio Fascione, Giuseppina Forgione, Daniela Sateriale, Caterina Pagliarulo

**Affiliations:** 1 Department of Diagnostic Services, Unit of Clinical Pathology, Marcianise Hospital, ASL Caserta, Viale Sossietta Scialla, 81025 Marcianise, Caserta, Italy; enza.mallardo@aslcaserta.it (E.M.); claudio.baliano@aslcaserta.it (C.A.B.); valeria.pedata@aslcaserta.it (V.P.); r.zinzi@studenti.unisannio.it (R.Z.); federica.mayella@aslcaserta.it (F.M.); mauro.murano@aslcaserta.it (M.M.); antonio.fascione@aslcaserta.it (A.F.); 2 Department of Science and Technology, University of Sannio, Via F. De Sanctis Snc, 82100 Benevento, Italy; gforgione@unisannio.it

**Keywords:** healthcare-associated infections (HAIs), hand hygiene practices, bloodstream infections, natural antimicrobial agents, multidrug-resistant pathogens, nosocomial infection control

## Abstract

Healthcare-associated infections (HAIs) remain a major public health concern, contributing to increased morbidity, mortality, and antimicrobial resistance. Healthcare workers (HCWs) are recognized as key vehicles in the transmission of nosocomial pathogens, primarily via contaminated hands and medical devices. This study assessed the effectiveness of hand hygiene protocols among HCWs, their correlation with bloodstream infections, and the potential of natural antimicrobial agents as complementary preventive measures. Between January and June 2025, 128 hand samples were collected from HCWs in surgical, intensive care, and internal medicine units of hospitals managed by ASL Caserta (Marcianise, n = 65; Piedimonte Matese, n = 30; Sessa Aurunca, n = 18; Maddaloni, n = 15). Sampling was performed upon entry to clinical areas and after antiseptic handwashing, using Rodac TSA plates to quantify microbial load (CFU/cm^2^). Isolates were identified via MALDI-TOF, and multidrug resistance was confirmed using the Phoenix BD system. Microbial growth was detected in 54.7% of samples. Coagulase-negative staphylococci, mainly *Staphylococcus epidermidis* and *S. hominis*, accounted for 67.1% of positive cultures, followed by Enterobacteriaceae (28.6%). Comparison with concurrently collected blood cultures revealed potential overlapping pathogens, with *Staphylococcus* spp. prevalence ranging from 35 to 56% and Gram-negatives from 18 to 39. Selected isolates were further tested for susceptibility to natural antimicrobial agents, derived from hop, red vine leaf, green tea, and pomegranate fruit, as well as thyme essential oil. Thyme essential oil (*Thymus vulgaris*) demonstrated notable antimicrobial activity, in several cases surpassing that of standard hygiene agents. These findings highlight not only that maintaining high standards of hand hygiene, proper care of invasive devices, and continuous microbiological surveillance is critical for preventing HAIs, but also that incorporating natural antimicrobial compounds into hygiene protocols may provide an effective and sustainable adjunct to reduce microbial contamination and combat infections caused by multidrug-resistant organisms.

## 1. Introduction

Healthcare-associated infections (HAIs), commonly referred to as nosocomial infections, are infections acquired during patient care in healthcare settings, including hospitals, long-term care facilities, and outpatient clinics. According to the European Centre for Disease Prevention and Control (ECDC), HAIs are generally defined as infections occurring at least 48 h after hospital admission, within 30 days following a surgical procedure, or up to 90 days in the presence of prosthetic implants [1]. HAIs represent a major global public health challenge, contributing to increased morbidity, mortality, prolonged hospital stays, and significant healthcare costs. Additionally, they play a key role in the dissemination of antimicrobial resistance (AMR), recognized by the World Health Organization (WHO) as one of the foremost threats to human health [2].

HAIs can affect multiple anatomical sites, with urinary tract infections (UTIs) associated with catheters, surgical site infections (SSIs), ventilator-associated pneumonia (VAP), bloodstream infections (BSIs), and device-related infections among the most frequent. The development of HAIs results from complex interactions between pathogenic microorganisms, patient susceptibility, and healthcare environmental factors [1,3,4]. Microorganisms may be endogenous, arising from the patient’s own microbiota, or exogenous, acquired from the healthcare environment, contaminated surfaces, medical devices, or the hands of healthcare workers (HCWs). Modern medical advances, including invasive procedures, widespread use of medical devices, and intensive antibiotic therapy, have improved patient survival but simultaneously increased HAI risk [4,5].

Epidemiological data indicate that HAIs predominantly occur in high-acuity settings such as intensive care units (ICUs), surgical wards, and internal medicine units. ICU patients, in particular, are frequently subjected to mechanical ventilation, vascular catheters, and other invasive procedures, significantly increasing infection risk. The ECDC reported that 3.7% of patients hospitalized in ICUs for more than two days develop a bloodstream infection, corresponding to over 90,000 patients annually across European ICUs, with approximately 4500 deaths directly attributable to bacteremia [1]. The recent 2022–2023 ECDC surveillance highlighted a 7% prevalence of HAIs in European acute care hospitals, with respiratory infections accounting for 29.3% of cases, UTIs 19.2%, SSIs 16.1%, and bloodstream infections 11.9% [6]. In Italy, national reports show similar trends, emphasizing the role of HAIs in antimicrobial resistance dissemination [7].

In healthcare settings, the spread of healthcare-associated infections (HAIs) results from complex interactions between pathogens, susceptible patients, and the hospital environment. Reservoirs include colonized or infected patients, healthcare workers (HCWs), medical devices, and environmental surfaces, all of which can facilitate pathogen persistence and dissemination. Transmission occurs predominantly through direct contact, especially via the hands of HCWs, followed by indirect contact with contaminated surfaces or medical devices, droplet exposure, and, less frequently, airborne spread. Droplet transmission generally occurs over short distances (<1 m), whereas airborne particles (<5 µm) can remain suspended for prolonged periods, increasing the risk in poorly ventilated areas [8,9]. Hand hygiene remains the most effective intervention to interrupt cross-transmission. HCWs can acquire transient microbiota from patients, surfaces, or devices, including Gram-positive organisms such as *Staphylococcus aureus* and coagulase-negative staphylococci (*S. epidermidis*), and Gram-negative pathogens like *Escherichia coli*, *Klebsiella pneumoniae*, *Pseudomonas aeruginosa*, and *Acinetobacter baumannii* [1,10]. Contaminated environmental surfaces further sustain HAIs, as pathogens can survive for hours to days on inanimate surfaces, acting as reservoirs for subsequent transmission [8]. Linking microbial isolates from environmental samples, healthcare workers’ hands, and clinical specimens such as blood cultures, even in the absence of genotyping data, is essential for better understanding the epidemiology of HAI transmission and for identifying potential cross-contamination pathways within healthcare settings.

The emergence of multidrug-resistant organisms (MDROs) represents a significant challenge in healthcare-associated infection (HAI) management. Methicillin-resistant *Staphylococcus aureus* (MRSA), vancomycin-resistant enterococci (VRE), and carbapenemase-producing Enterobacteriaceae (CPE) are associated with increased morbidity, limited therapeutic options, prolonged hospital stays, and higher healthcare costs [7]. Effective prevention and control strategies must integrate infection prevention measures, antimicrobial stewardship, and surveillance systems to monitor resistance trends and guide interventions. The growing prevalence of high-risk, multidrug-resistant pathogens underscores the need for continuous microbiological surveillance, as monitoring environmental contamination and comparing isolates from patients and healthcare workers can guide targeted infection control strategies. Continuous microbiological monitoring is essential for HAI prevention, allowing assessment of pathogen prevalence and environmental contamination. Sampling of surfaces and healthcare worker hands using standardized techniques, such as ISO 18593:2004, supports targeted interventions and evaluation of cleaning protocols [8,11]. Comparing environmental isolates with clinical specimens can reveal epidemiological links and inform infection control strategies. Such integrated approaches facilitate evidence-based decisions and contribute to patient safety.

Traditional chemical disinfectants, while effective, can lead to resistance selection, environmental impact, and potential toxicity to patients and staff. Consequently, interest has grown in natural and sustainable alternatives, including polyphenol-rich plant extracts (e.g., from *Camellia sinensis*, *Vitis vinifera*, *Punica granatum*, *Olea europaea*) and essential oils (EOs), such as *Thymus vulgaris* EO. These agents exert antimicrobial, antifungal, and antiviral effects through mechanisms including protein denaturation, membrane disruption, metal chelation, and biofilm inhibition, and may complement conventional hygiene protocols [12,13,14].

The aim of this study is to evaluate the effectiveness of current infection prevention and control (IPC) protocols in limiting HAIs and to explore innovative, natural antimicrobial strategies as complementary interventions. By integrating environmental and clinical microbiological data, this work seeks to identify critical contamination sources, characterize pathogen transmission pathways, and provide evidence for the implementation of novel, sustainable disinfection approaches. The originality of this study lies in its integrated approach combining microbiological surveillance, assessment of hand and environmental surface contamination, and correlation with nosocomial bloodstream infections, with the evaluation of natural antimicrobial agents as potential alternative preventive strategies against pathogens in high-risk hospital settings.

## 2. Materials and Methods

### 2.1. Study Design

This study was conducted at the Microbiology Laboratory of the Clinical Pathology Unit of Marcianise Hospital, part of the Local Health Authority (ASL) of Caserta, southern Italy, one of the major referral centers in the region, providing tertiary-level surgical and medical care, which makes it a critical site for evaluating infection control practices. Microbiological sampling activities were carried out between January and June 2025 and involved multiple hospitals within the ASL Caserta network, including Marcianise, Sessa Aurunca, Piedimonte Matese, and Maddaloni. Environmental sampling was performed on operating room surfaces at Marcianise Hospital. In parallel, microbiological monitoring of healthcare workers’ (HCWs) hands was conducted in intensive care units and internal medicine wards across all participating hospitals. During the same period, positive blood cultures collected from these clinical units were also analyzed to investigate potential correlations between environmental contamination, hand colonization, and bloodstream infections. Subsequently, selected microorganisms isolated from environmental surfaces and HCWs’ hands were screened for susceptibility to natural substances at the Microbiology Laboratory of the University of Sannio.

### 2.2. Microbiological Hygiene Monitoring

#### 2.2.1. Environmental Surface Sampling

Environmental microbiological investigations were carried out in the operating rooms of Marcianise Hospital to assess microbial contamination on critical surfaces. Sampling procedures were performed in accordance with ISO 18593:2018 for microbiological monitoring in healthcare environments [15]. Surfaces were selected based on their frequency of contact and clinical relevance within the operating room setting. In particular, five sampling locations were included: operating table, medication table, multiparameter monitor, surgical lamp, ventilation grille. Each site was sampled using RODAC (Replicate Organism Detection and Counting) contact plates (Liofilchem S.r.l., Roseto degli Abruzzi, Italy) with a diameter of 55 mm, corresponding to a sampling surface of 16 cm^2^, containing Tryptic Soy Agar supplemented with neutralizing agents to inactivate residual disinfectants. The plates were applied directly onto dry surfaces for approximately 10 s using uniform pressure to ensure adequate contact. Sampling was carried out 30 to 60 min after routine sanitation procedures in order to allow sufficient time for disinfectant activity and to obtain results representative of real operating conditions. After collection, the plates were transported under sterile conditions to the microbiology laboratory for subsequent incubation and analysis.

#### 2.2.2. Hand Sampling of Healthcare Workers (HCWs)

Microbiological monitoring of HCWs’ hands was performed to evaluate residual contamination following hand hygiene procedures and to assess the potential role of HCWs in the transmission of microorganisms. The study involved HCWs working in intensive care units and internal medicine wards across all participating hospitals, as well as operating room staff at Marcianise Hospital. In total, 128 microbiological samples were collected from HCWs’ hands during the study period. Sampling was performed using the same plates and methodology described for environmental surface sampling. During the procedure, HCWs were asked to gently press the fingertips of the three central fingers onto the agar surface for approximately 10 s, applying light and uniform pressure. The procedure was performed, by trained operators to ensure consistency of contact modality across all samples, in accordance with standardized hospital microbiological surveillance procedures and occupational monitoring ISO guidelines [15]. Samples were collected both before the hand hygiene procedures and after surgical handwashing followed by sterile drying, allowing the assessment of both qualitative and quantitative residual microbial contamination.

#### 2.2.3. Quantification of Microbial Contamination

All collected plates were incubated aerobically at 37 ± 1.5 °C for 24 to 48 h. After incubation, the developed colonies were counted and used to quantify microbial contamination. Results for environmental samples were expressed as colony-forming units (CFU) per plate, whereas for hand samples the microbial load was expressed as colony-forming units per square centimeter (CFU/cm^2^), calculated by dividing the number of colonies by the sampled surface area of 16 cm^2^. The results were interpreted according to reference thresholds defined by national guidelines (ISO 18593:2018). In brief, for environmental samples, values between 5 and 15 CFU per plate were considered acceptable, while for HCWs’ hands the acceptable limit was set at 0.5 CFU/cm^2^, whereas higher values indicated the need to review sanitation procedures [15].

#### 2.2.4. Microbial Identification of Environmental and Hand Isolates by MALDI-TOF MS

Microbial colonies obtained from culture plates were identified using matrix-assisted laser desorption/ionization time-of-flight mass spectrometry (MALDI-TOF MS) with the VITEK MS PRIME system (BioMérieux, Marcy-l’Étoile, France). All morphologically distinct colonies grown on selective and differential culture media routinely used in clinical microbiology were considered for analysis. Following incubation, colony morphology was systematically assessed, recording size, pigmentation, surface texture, elevation, and margin characteristics. When multiple colonies were present on the same plate, representative colonies exhibiting distinct morphological features were selected for identification. For identification, a small amount of colony material was transferred onto a target plate and overlaid with α-cyano-4-hydroxycinnamic acid matrix. After drying at room temperature, the samples were subjected to laser ionization. The generated ions were separated based on their mass-to-charge ratio, producing a characteristic mass spectrum primarily derived from ribosomal proteins. The resulting spectra were automatically compared with reference profiles stored in the system database, allowing rapid identification of microorganisms at the species level. The detection of potentially pathogenic microorganisms such as *Staphylococcus aureus*, coagulase-negative staphylococci, Enterobacteriaceae, *Pseudomonas* spp., or *Aspergillus* spp. was considered indicative of inadequate hygiene or disinfection practices.

### 2.3. Blood Culture Monitoring

#### 2.3.1. Automated Blood Culture Analysis

During the study period, blood cultures were analyzed to evaluate possible correlations with environmental and hand isolates. Samples were processed using the BD BACTEC FX system (Becton Dickinson, Franklin Lakes, NJ, USA), which detects microbial growth through continuous monitoring of carbon dioxide production by means of fluorescent sensors. Blood samples were inoculated into specific culture bottles designed for aerobic (BD BACTEC Plus Aerobic), anaerobic (BD BACTEC Lytic Anaerobic), and fungal (BD BACTEC Mycosis IC/F) growth and incubated at 35 ± 1.5 °C under continuous agitation to enhance microbial proliferation. When microbial growth was detected, the system flagged the bottle as positive, prompting further microbiological analyses.

#### 2.3.2. Gram Staining

Gram staining was performed on positive blood culture samples to provide rapid preliminary classification of microorganisms. Smears were prepared, air-dried, heat-fixed, and stained according to the standard Gram protocol using an automated staining system (BASO AS-316GT, Baso Diagnostics, Zhuhai, China). Microscopic examination under oil immersion allowed differentiation between Gram-positive and Gram-negative bacteria, as well as evaluation of cellular morphology and arrangement. These findings provided preliminary diagnostic information to guide subsequent analyses.

#### 2.3.3. Molecular Identification

Rapid molecular identification was performed using the FilmArray Blood Culture Identification (BCID) panel (BioFire Diagnostics, bioMérieux, Salt Lake City, UT, USA), a multiplex PCR-based system capable of detecting a wide range of Gram-positive and Gram-negative bacteria, yeasts, and selected antimicrobial resistance genes. A small aliquot from positive blood culture bottles was loaded into a single-use pouch containing all necessary reagents. The system automatically performed nucleic acid extraction, amplification, and detection. Results were available within approximately one hour, enabling early microbiological characterization of bloodstream infections.

#### 2.3.4. Microbial Identification of Blood Culture Isolates by MALDI-TOF MS

Positive samples were subcultured on appropriate solid media and incubated at 37 ± 1.5 °C for 18 to 24 h to obtain isolated colonies, which were then identified using MALDI-TOF MS as previously described in paragraph 2.3.4. Microorganisms isolated from blood cultures were subsequently compared with those obtained from environmental surfaces and HCWs’ hands to identify potential epidemiological correlations and transmission pathways.

### 2.4. Antimicrobial Susceptibility Screening

#### 2.4.1. Bacterial Isolates

To explore the innovative potential of natural extracts as complementary sanitizing agents to be integrated into conventional hospital hand hygiene and environmental disinfection protocols, their antimicrobial activity was evaluated against microorganisms associated with healthcare-associated infections (HAIs).

For this purpose, bacterial strains isolated from the hands of healthcare workers were selected, representing both Gram-positive and Gram-negative species commonly involved in nosocomial transmission dynamics. In particular, the study included the following bacterial isolates: *Staphylococcus epidermidis* HSSe1, *Staphylococcus hominis* HSSh1, *Staphylococcus aureus* HSSa1, and *Klebsiella pneumoniae* HSKp1. Species-level identification was confirmed using matrix-assisted laser desorption/ionization time-of-flight mass spectrometry (MALDI-TOF MS) with the VITEK MS Prime system (bioMérieux, Marcy-l’Étoile, France). Identification was based on characteristic protein spectral fingerprints, and a confidence value of 99.9% was obtained for *all* isolates, representing the maximum score achievable by the system. The corresponding antimicrobial susceptibility profiles of selected bacterial isolates are reported in the Supplementary Material (Tables S1 and S2).

#### 2.4.2. Selected Antimicrobial Agents

Polyphenolic extracts were provided by EPO Istituto Farmochimico Fitoterapico S.r.l. (Milan, Italy).

The hop extract (HLE, *Humulus lupulus* extract) is a dry hydroalcoholic extract obtained from *Humulus lupulus* L. (Cannabaceae) (CAS: 8060-28-4; 8016-25-9), derived from strobiles (female inflorescences) collected during flowering (August–September) through ethanol/water extraction and standardized to flavonoids expressed as rutin (≥0.4%), used as a biological marker; humulone, lupulone, essential oil, and tannins are also present.

The red vine leaf extract (GLE, *Vitis vinifera* leaf extract) is a dry aqueous extract obtained from *Vitis vinifera* L. (Vitaceae) (CAS: 84929-27-1), derived from leaves harvested in autumn, extracted with water and characterized by a total polyphenol content ≥ 5.0% (*w*/*w*); flavonoids, tannins, and anthocyanins are also present.

The green tea extract (GTE, *Camellia sinensis* extract) is a dry hydroalcoholic extract obtained from *Camellia sinensis* (L.) Kuntze (Theaceae) (CAS: 84650-60-2), derived from leaves collected between March and July through ethanol/water extraction and characterized by a high content of polyphenols (≥95%) and catechins (≥65%), with epigallocatechin gallate (EGCG) used as a biological marker; methylxanthines (caffeine, theobromine, theophylline), flavonoids, tannins, chlorogenic acid, saponins, vitamins, and minerals are also present.

The pomegranate fruit extract (PFE, *Punica granatum* extract) is a dry hydroalcoholic extract obtained from *Punica granatum* L. (Punicaceae) (CAS: 84961-57-9), derived from ripe fruits through ethanol/water extraction and characterized by a total polyphenol content ≥ 20% and ellagic acid ≥ 10%, used as a biological marker; punicalagins are also present.

Thyme essential oil (TEO, Thyme Oil—White FG, W306509, Sigma-Aldrich, Merck, Darmstadt, Germany) is an essential oil obtained from *Thymus vulgaris* L. and/or *Thymus zygis* (Lamiaceae).

All polyphenolic extracts were tested after preparation of a stock solution at a defined concentration of 20 mg/mL, whereas thyme essential oil was used in its pure form. The antimicrobial agents were subsequently adjusted to the final working concentrations used in the antimicrobial assays, as described in the corresponding methodological sections.

#### 2.4.3. Agar Well Diffusion Method

To qualitatively evaluate the antimicrobial activity of TEO and the polyphenolic extracts (HLE, GLE, GTE, PFE) against the selected clinical isolates, a preliminary in vitro screening was performed using the agar well diffusion assay, as described by Sateriale et al. [16], with minor modifications. Briefly, aliquots of each microbial suspension, adjusted to an optical density (O.D.) of 0.5 at 600 nm, were spread onto Luria–Bertani (LB) agar plates (HiMedia GmbH, Odenwaldstr, Modautal, Germany). Wells of 6 mm diameter were then aseptically punched into the agar at appropriate distances using sterile Pasteur pipettes (Sigma-Aldrich S.r.l., Milan, Italy). Aliquots of TEO (20 µL) and each extract (1 mg/well) were dispensed into the wells. After incubation at 37 ± 2 °C for 24 h, the diameter (mm) of the inhibition zones formed around the wells was measured [17]. Antimicrobial activity was expressed as the mean diameter of inhibition zones (MDIZ) against the tested microorganisms. A 2% (*w*/*v*) sodium percarbonate (SPC) (Sigma-Aldrich, Merck, Darmstadt, Germany) solution (50 µL) was used as a positive control. All experiments were performed in triplicate on independent cultures.

#### 2.4.4. Tube Dilution Method

The susceptibility of clinical isolates was determined using the broth microdilution method with a standardized inoculum of 1 × 10^5^ CFU/mL (colony-forming units/mL), in accordance with the guidelines of the Clinical and Laboratory Standards Institute [18], using sodium percarbonate as a positive control. The minimum inhibitory concentration (MIC) was defined as the lowest concentration of the antimicrobial agent capable of inhibiting visible microbial growth. The minimum bactericidal concentration (MBC) was defined as the lowest concentration of the antimicrobial agent required to kill 99% of the initial bacterial inoculum.

### 2.5. Statistical Data Analysis

Data were analyzed and graphically represented using GraphPad Prism version 8.0 (GraphPad Software, San Diego, CA, USA). Statistical significance was evaluated using one-way analysis of variance (ANOVA), followed by Dunnett’s and Tukey’s post hoc tests for multiple comparisons, as appropriate. In addition, two-way ANOVA with Bonferroni post hoc correction was applied where indicated. A *p*-value < 0.05 was considered statistically significant in all analyses.

## 3. Results

### 3.1. Environmental Surface Contamination

A total of 27 microbiological samples were collected from high-touch surfaces within the operating rooms of Marcianise Hospital between January and June 2025. The sampled sites included the operating table, medication table, multiparameter monitor, surgical lamp, and ventilation grille. Samples were classified as positive when microbial growth exceeded the accepted threshold of 15 CFU per plate, in accordance with recommended environmental hygiene standards for critical care areas.

**Figure 1:**
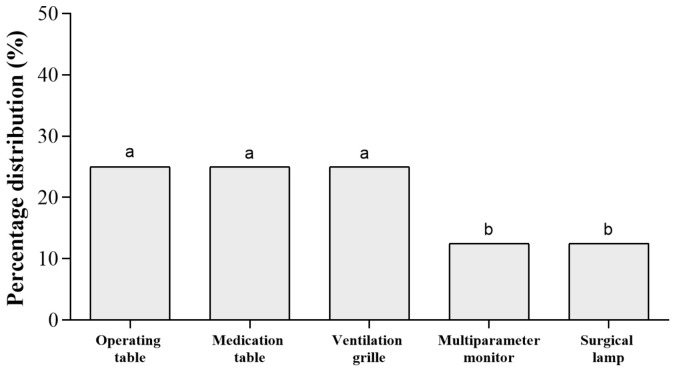
Percentage distribution of positive microbiological samples collected from critical high-touch surfaces in operating rooms, including operating table, medication table, ventilation grille, multiparameter monitor, and surgical lamp. Samples were considered positive when microbial growth exceeded the threshold of 15 CFU per plate. Data were analyzed using one-way analysis of variance (ANOVA), followed by Tukey’s post hoc test for multiple comparisons among surfaces (*p* < 0.05). Different letters (a, b) indicate statistically significant differences between groups; surfaces sharing the same letter are not significantly different.

Overall, 29.6% of the samples exceeded the contamination threshold, indicating a non-negligible level of environmental bioburden within the operating theater. The highest rates of contamination were observed on the operating table, medication table, and ventilation grille, each accounting for 25% of positive samples, while the multiparameter monitor and surgical lamp each showed a positivity rate of 12.5% (Figure 1).

Microbiological identification by MALDI-TOF analysis revealed that the isolated microorganisms predominantly belonged to the commensal skin flora. *Staphylococcus epidermidis* and *Staphylococcus warneri* were the most frequently detected species, while *Micrococcus luteus* was isolated primarily from the ventilation grille (Table 1). Although these organisms are generally considered of low pathogenicity, their recovery above recommended thresholds is of clinical relevance, as it indicates persistent environmental contamination and a potential reservoir for indirect transmission.

### 3.2. Microbial Contamination of Healthcare Workers’ Hands

**Figure 2:**
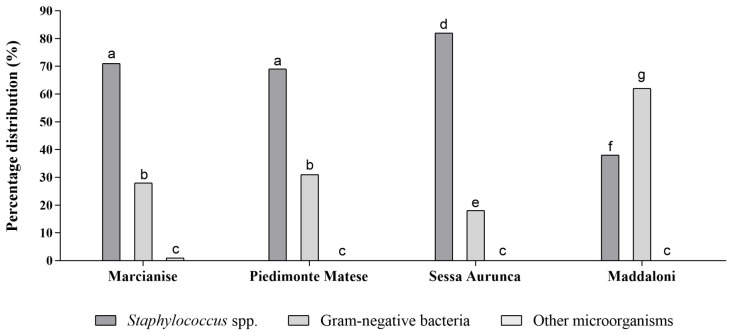
Percentage distribution of microorganisms isolated from healthcare workers’ hands across different hospital facilities. Data were analyzed using two-way analysis of variance (ANOVA), followed by Bonferroni’s post hoc test for multiple comparisons. Different letters (a–g) indicate statistically significant differences between groups (*p* < 0.05).

Between January and June 2025, a total of 128 microbiological samples were collected from the hands of healthcare personnel working in the operating rooms, intensive care units, and internal medicine wards across different hospitals of the ASL of Caserta. Samples were analyzed to assess the presence of residual microbial contamination following hand hygiene procedures and to evaluate the potential role of healthcare workers as vehicles in microbial transmission dynamics. The distribution of positive samples and the identified microorganisms across the different hospital facilities is reported in Figure 2 and Table 2.

Overall, 54.7% of the samples (70/128) exceeded the acceptable threshold of 0.5 CFU/cm^2^ for hand contamination, as defined by national and international hygiene guidelines (ISO 18593:2018), while the remaining 45.3% fell within the recommended safety limits. This finding indicates that, despite the implementation of hand hygiene procedures, a substantial proportion of healthcare workers still presented measurable microbial contamination. Among positive samples, a clear predominance of coagulase-negative staphylococci was observed, particularly *Staphylococcus epidermidis* and *Staphylococcus hominis*, which together accounted for 67.1% of all isolates. These organisms represent typical components of the resident skin microbiota; however, their persistence after hygiene procedures may reflect incomplete decontamination or rapid recontamination during clinical activity. A lower proportion (28.6%) of isolates consisted of Gram-negative bacteria belonging to the Enterobacteriaceae family, suggesting transient contamination likely acquired through patient or environmental contact. Additional sporadic isolates included *Staphylococcus aureus, Staphylococcus haemolyticus, Staphylococcus warneri*, *Staphylococcus pseudintermedius*, and *Bacillus pumilus*. All microorganisms were identified at species level using MALDI-TOF mass spectrometry, ensuring rapid and accurate taxonomic classification.

The highest number of positive samples was recorded in the Marcianise hospital, followed by Piedimonte Matese, Sessa Aurunca, and Maddaloni (Table 2). This distribution suggests variability in contamination levels across healthcare settings, potentially reflecting differences in workload intensity, compliance with hand hygiene protocols, or local infection control practices.

### 3.3. Bloodstream Infection Surveillance

**Figure 3:**
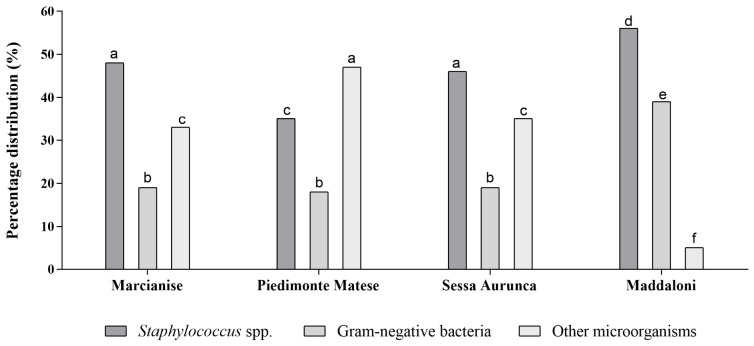
Percentage distribution of the main microorganisms isolated from blood cultures across hospital facilities. Data were analyzed using Two-way analysis of variance (ANOVA), followed by Bonferroni’s post hoc test for multiple comparisons. Different letters (a–f) indicate statistically significant differences between groups (*p* < 0.05).

Between January and June 2025, a total of 525 positive blood cultures were analyzed across the participating hospitals of the ASL of Caserta, distributed as follows: Marcianise (n = 171), Piedimonte Matese (n = 150), Sessa Aurunca (n = 104), and Maddaloni (n = 100). For the purposes of this study, only blood culture isolates potentially comparable to those recovered from environmental and hand sampling were included, in order to explore a possible epidemiological link and assess the putative role of healthcare workers’ hands in pathogen transmission. Overall, the microbiological profile of bloodstream isolates showed a predominance of *Staphylococcus* spp., followed by Gram-negative bacteria, with a residual proportion of other microorganisms. The distribution of isolates across hospital facilities is reported in Figure 3 and Table 3.

*Staphylococcus* spp. accounted for approximately 35.0% to 56.0% of isolates depending on the hospital setting, while Gram-negative bacteria ranged from 18.0% to 32.2%. The remaining fraction consisted of other microorganisms, which were particularly relevant in Sessa Aurunca and Maddaloni, where they represented a substantial proportion of positive blood cultures.

A comparative evaluation of microbiological profiles between bloodstream isolates and those recovered from hand and environmental samples revealed a substantial overlap. In both settings, *Staphylococcus* spp. represented the dominant group, accompanied by a consistent presence of Gram-negative bacteria.

### 3.4. Antimicrobial Activity of Natural Compounds

**Figure 4:**
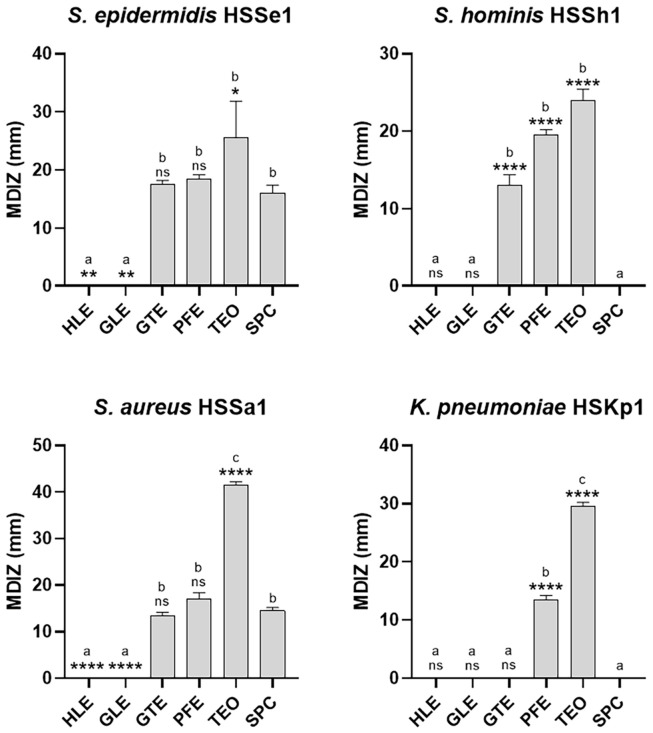
In vitro antibacterial activity of HLE, GLE, GTE, PFE, TEO, and SPC evaluated using the agar well diffusion method against colonizing bacterial strains isolated from healthcare workers: *S. epidermidis* HSSe1, *S. hominis* HSSh1, *S. aureus* HSSa1, and *K. pneumoniae* HSKp1. The mean diameter of the inhibition zone (mm) is reported as the mean of values obtained from assays performed in triplicate ± standard deviation. Statistical significance was assessed using one-way ANOVA followed by Dunnett’s correction (*p* < 0.05) for comparison with the positive control, and one-way ANOVA followed by Tukey’s correction (*p* < 0.05) for multiple comparisons. Asterisks indicate statistical significance compared with the positive control (**** *p* < 0.0001; ** *p* < 0.01; * *p* < 0.05); the absence of asterisks indicates no significance. Letters (a–c) indicate statistically significant differences among values; results not significantly different share the same letter. MDIZ: mean diameter of inhibition zone; HLE, hop extract; GLE, red vine leaf extract; GTE, green tea extract; PFE, pomegranate extract; TEO, thyme essential oil; SPC, sodium percarbonate.

The antibacterial activity of the tested treatments was evaluated using the agar diffusion assay (Figure 4). The results revealed significant differences depending on the chemical nature of the agents and the structural characteristics of the microorganisms. Overall, a gradient of efficacy was observed: thyme essential oil (TEO) showed the highest activity, followed by pomegranate extract (PFE) and, to a lesser extent, green tea extract (GTE), whereas hop extract (HLE), red vine leaf extract (GLE), and SPC exhibited limited or strain-specific activity. Among Gram-positive bacteria, *Staphylococcus* strains showed overall greater susceptibility compared to Gram-negative bacteria, including their response to the positive control. In *S. epidermidis*, GTE and PFE produced moderate inhibition zones of 17.50 ± 0.71 mm and 18.50 ± 0.71 mm, respectively, whereas TEO induced significantly greater inhibition (25.50 ± 6.34 mm). SPC showed intermediate activity (16.00 ± 1.41 mm), lower than PFE and markedly lower than TEO, indicating reduced effectiveness of the oxidative mechanism compared to the most active natural compounds. HLE and GLE were inactive. Similarly, *S. hominis* exhibited greater overall susceptibility to natural treatments than to the positive control. In this case, TEO was confirmed as the most effective treatment (24.00 ± 1.41 mm), followed by PFE (19.50 ± 0.71 mm) and GTE (13.00 ± 1.41 mm). SPC did not produce any measurable inhibition zone, suggesting marked tolerance to oxidative stress. In *S. aureus*, all treatments showed antibacterial activity, with substantial differences. TEO induced the largest inhibition zone (41.50 ± 0.71 mm), indicating high strain sensitivity. PFE and GTE showed intermediate activity (17.00 ± 1.41 mm and 13.50 ± 0.71 mm, respectively), whereas HLE and GLE were weakly active. SPC exhibited a lower inhibitory effect than PFE and significantly lower than TEO, resulting comparable to the less effective extracts. In *K. pneumoniae*, a Gram-negative bacterium, an overall reduced susceptibility was observed, including a poor response to the positive control. TEO produced significant inhibition (29.50 ± 0.71 mm), followed by PFE (13.50 ± 0.71 mm), whereas SPC, GTE, HLE, and GLE were ineffective.

The comparative analysis with respect to SPC showed that TEO exhibited not only the broadest spectrum of activity but also superior antibacterial potency against all tested strains. PFE was the second most effective treatment, whereas GTE showed lower or comparable activity to SPC. HLE and GLE, being inactive, were excluded from the determination of minimum inhibitory concentration (MIC) and minimum bactericidal concentration (MBC). The quantitative evaluation of in vitro antibacterial activity (Table 4) confirmed that GTE and PFE mainly exerted a bacteriostatic effect, with MIC values ranging from 1 to 20 mg mL^−1^ and MBC values often >20 mg mL^−1^. In contrast, TEO showed consistently very low MIC values (0.25 µL mL^−1^) against Gram-positive strains (*S. epidermidis*, *S. hominis*, *S. aureus*), with MBC values ranging from 1 to 2.5 µL mL^−1^, indicating marked bactericidal activity. In Gram-negative bacteria, TEO maintained good activity, with a MIC value of 2 µL mL^−1^ in *K. pneumoniae*, while higher MBC values (5 µL mL^−1^) confirmed the difficulty in achieving a complete bactericidal effect. In comparison, GTE and PFE showed limited or absent bactericidal activity (>20 mg mL^−1^). TEO exhibited strong antimicrobial activity against all tested strains, with MIC values ranging from 0.25 to 2 μL mL^−1^. Staphylococcal isolates showed high susceptibility to TEO, including strains resistant to multiple antibiotics such as oxacillin, erythromycin, clindamycin, and tetracycline (Table S1). Similarly, the *K. pneumoniae* HSKp1 strain, resistant to several conventional antibiotics (Table S2), remained susceptible to TEO. Overall, TEO displayed antimicrobial efficacy comparable to, or greater than, that of several tested antibiotics.

Overall, the data indicate that TEO exhibits the most promising antimicrobial profile among the tested compounds, characterized by low MIC values, significant bactericidal activity, and a broad spectrum of action, suggesting its potential application as a natural antimicrobial agent for controlling bacterial colonization and preventing healthcare-associated infections.

## 4. Discussion

The present study reinforces the concept that microbial contamination of high-risk hospital environments, particularly operating rooms and intensive care settings, represents a critical determinant in the transmission dynamics of healthcare-associated infections (HAIs). Although the microbiological profile observed was predominantly composed of commensal skin flora, the sustained presence of microorganisms on critical surfaces and on the hands of healthcare workers, even at low levels, highlights a tangible risk of cross-contamination with direct implications for patient safety.

These findings are consistent with the well-established role of the hospital environment as an interconnected ecosystem in which surfaces, patients, and healthcare personnel continuously interact, facilitating microbial exchange. High-touch surfaces, in particular, have been repeatedly identified as potential reservoirs of microorganisms capable of persisting despite routine cleaning procedures, thereby contributing to indirect transmission pathways [19]. In this context, the present results support the view that even organisms belonging to the resident skin microbiota may acquire clinical relevance when environmental persistence and repeated contact opportunities are present. Although coagulase-negative staphylococci represented the predominant isolates, reflecting the expected cutaneous microbiota, their clinical relevance should not be underestimated, as they are recognized opportunistic pathogens, particularly in patients with indwelling medical devices [10]. The detection of Gram-negative bacteria, instead, is suggestive of transient contamination acquired during care activities, consistent with their known role as environmentally associated organisms involved in nosocomial transmission pathways.

The comparative analysis between environmental samples, hand swabs from healthcare workers, and blood cultures revealed similarities in the microbiological profiles observed across the different sample types. These findings may be consistent with a potential cross-contamination network within the healthcare environment, in which healthcare workers’ hands could represent one of several possible vehicles of transmission. This concordance suggests a potential epidemiological relationship between environmental contamination, hand carriage, and bloodstream infections, supporting the hypothesis that inadequate hand hygiene and environmental persistence may contribute to pathogen transmission within hospital settings. It is important to note that, in the absence of molecular typing methods such as Multilocus Sequence Typing (MLTS) or Whole-Genome Sequencing (WGS), the present study cannot establish clonal relatedness among isolates recovered from different sources. Therefore, the observed overlap in bacterial species observed among environmental, hand, and clinical samples should not be interpreted as evidence of direct transmission events or a common source. Instead, these findings should be considered as an epidemiological and microbiological association consistent with a plausible cross-contamination network within the healthcare environment. The results suggest a possible epidemiological association among these reservoirs and are consistent with established transmission dynamics in healthcare settings, where healthcare workers are recognized contributors to cross-contamination. This interpretation is supported by the well-documented role of healthcare workers’ hands as a primary vehicle for cross-transmission of microorganisms in clinical settings [9,20].

The lack of molecular typing analysis prevented the assessment of genetic relatedness among microorganisms isolated from environmental surfaces, healthcare worker hands, and blood cultures, representing the most significant limitation of this study. Consequently, the observed overlap in bacterial species cannot be interpreted as evidence of clonal transmission, but only as a possible epidemiological association. Future investigations incorporating molecular typing techniques, such as whole-genome sequencing, together with a broader sampling strategy, would provide a more comprehensive understanding of pathogen circulation, strain relatedness, and potential transmission dynamics within healthcare settings.

A further limitation of the present study is the absence of bioaerosol sampling, which did not allow the assessment of airborne microbial load within the investigated healthcare environments. Although direct-contact transmission remains a major route for the dissemination of healthcare-associated pathogens, surface contamination in high-risk settings should also be interpreted in the context of bioaerosol deposition. In operating rooms and intensive care units, microbial particles generated by patients, healthcare workers, respiratory activities, skin shedding, staff movement, and aerosol-generating procedures may remain airborne and subsequently settle onto environmental surfaces [21,22]. Experimental studies and computational fluid dynamics (CFD) models have demonstrated that airborne microorganisms can contribute to both air and surface contamination, supporting their potential role in cross-transmission pathways [23,24]. The continuous settling of pathogen-laden aerosols may lead to rapid recontamination of high-touch surfaces, even after cleaning and disinfection, thereby sustaining persistent environmental reservoirs of healthcare-associated pathogens [25]. Moreover, the prolonged survival of several nosocomial microorganisms on inanimate surfaces further reinforces the epidemiological relevance of these reservoirs [26]. Airborne microbial transport and surface deposition are strongly influenced by environmental and engineering factors, including ventilation design, air-exchange rates, airflow patterns, pressure differentials, and filtration efficiency [27,28]. In this regard, heating, ventilation, and air conditioning (HVAC) performance and high-efficiency particulate air (HEPA) filtration systems may play a crucial role in controlling airborne microbial loads and limiting microbial settling onto surfaces [28,29]. Conversely, inadequate ventilation or filtration may promote the accumulation of airborne contaminants and increase surface microbial burden through repeated bioaerosol deposition [22]. Although HVAC operational parameters were not evaluated in the present study, their potential influence should be considered when interpreting environmental contamination patterns. Future studies should integrate surface microbiological surveillance with indoor air-quality assessments, including airborne microbial monitoring, ventilation performance evaluation, air-exchange rate assessment, and filtration efficiency analysis. Such an integrated approach may provide a more comprehensive understanding of pathogen circulation within healthcare environments and support more effective infection prevention and control (IPC) strategies [28,29].

From a preventive perspective, these findings underline that the implementation of infection control protocols is insufficient to ensure optimal safety outcomes if not accompanied by rigorous adherence and continuous verification. Systematic monitoring, particularly through microbiological surveillance, represents a key tool for identifying critical points, evaluating intervention effectiveness, and guiding corrective actions. In this framework, structured risk management activities play a central role in integrating surveillance data into continuous improvement processes [30]. In addition, the results highlight the importance of strengthening training programs for healthcare workers and ensuring ongoing education aimed at improving compliance with hand hygiene and environmental sanitation procedures. The implementation of objective auditing systems and performance indicators is essential to reduce variability in practice and enhance adherence to evidence-based protocols. These considerations are consistent with international recommendations advocating multimodal infection prevention strategies that integrate behavioral, organizational, and technical interventions [31,32].

Within this context, the role of new disinfectants and alternative antimicrobial strategies becomes particularly relevant. Disinfectants containing clorexidine and sodium percarbonate (SPC) have been developed as effective and relatively safe agents for the elimination of viruses and bacteria in both animal and human settings; however, it is well recognized that most chemical disinfectants may still pose risks to living organisms and the environment [33]. This issue reinforces the need for innovative approaches that can complement or partially replace conventional disinfectants, improving both efficacy and safety profiles. The present findings indicate that the limited activity of SPC against Gram-negative bacteria may be attributed to the inability of oxidative mechanisms to overcome the structural barrier of the outer membrane. In contrast, *Thymus vulgaris* essential oil (TEO) demonstrated a markedly higher antimicrobial efficacy, consistent with its lipophilic nature and its ability to target bacterial membranes directly. Essential oils exert their antimicrobial activity primarily through interactions with lipid bilayers, where their components insert between phospholipids, disrupting membrane permeability and fluidity and ultimately compromising cellular integrity [34]. In particular, thymol, the major constituent of TEO, plays a central role in its antibacterial activity by destabilizing both inner and outer bacterial membranes, promoting leakage of intracellular macromolecules, including DNA. In parallel, TEO and thymol induce intracellular accumulation of reactive oxygen species (ROS), resulting in oxidative stress and DNA damage. Furthermore, thymol may directly interact with bacterial DNA, interfering with its physiological functions and contributing to bacterial cell death [35]. These multifaceted mechanisms are consistent with broader evidence describing essential oils as effective antimicrobial agents against a wide range of clinically relevant pathogens, including antibiotic-resistant strains [36]. Beyond direct bactericidal effects, essential oils have also been shown to disrupt bacterial membrane proteins and increase cellular permeability, thereby enhancing antimicrobial efficacy. Importantly, they may also interfere with early stages of biofilm formation by inhibiting bacterial adhesion or disrupting quorum sensing systems, which regulate biofilm development, virulence factor expression, and antimicrobial resistance mechanisms [37]. These properties are particularly relevant in hospital environments, where biofilm-associated persistence represents a major challenge for infection control. Overall, TEO emerged as the most effective agent in terms of antimicrobial spectrum and bacteriostatic activity, followed by plant-derived phenolic extracts, whereas green tea extract showed limited or comparable efficacy to SPC. Hydroalcoholic extracts did not demonstrate significant activity. These findings suggest that plant-derived antimicrobials may represent a promising complementary strategy to conventional disinfection protocols, particularly in settings characterized by high microbial pressure and elevated risk of HAIs. With regard to potential applications in hospital contamination control and infection prevention and control strategies, these compounds could potentially be incorporated into hand hygiene formulations (e.g., hand antiseptics and hand rubs), surface disinfectants, antimicrobial wipes, or protective coating systems for high-touch hospital surfaces, where microbial transmission is particularly critical. In particular, essential oils and plant-derived polyphenols have been investigated as complementary ingredients in sanitizing and decontamination products due to their broad-spectrum antimicrobial activity and natural origin, and are considered Generally Recognized as Safe (GRAS) [38,39]. Nevertheless, the translation of in vitro antimicrobial activity into real-world healthcare and clinical settings requires caution, as factors such as formulation stability, compatibility with existing disinfectants and hospital-grade products, skin tolerability, toxicity, efficacy under practical use conditions, and the presence of organic load in hospital environments may significantly influence their performance in clinical infection control applications [36]. Previous studies have explored the incorporation of essential oils and plant extracts into antimicrobial and antiseptic formulations for healthcare, environmental disinfection, and surface decontamination systems, reporting encouraging results, including preliminary evaluations in simulated or in vivo conditions [40,41]. However, additional in vivo studies and clinical trials are required before these compounds can be recommended for routine use in hospital infection prevention and control programs.

## 5. Conclusions

In conclusion, the management of microbial contamination in healthcare environments requires a comprehensive and integrated strategy that combines rigorous hand hygiene, effective environmental cleaning, continuous education of healthcare workers, systematic microbiological surveillance, and the adoption of innovative antimicrobial approaches. The integration of these measures within structured infection prevention and risk management frameworks may significantly contribute to reducing the burden of healthcare-associated infections and improving both patient and healthcare worker safety.

The findings of this study suggest a potential epidemiological association among environmental contamination, healthcare workers’ hand colonization, and clinical isolates within the investigated settings. However, these observations cannot be interpreted as evidence of clonal transmission but rather as a plausible cross-contamination network that warrants further investigation using high-resolution genomic approaches.

In addition, the incorporation of natural antimicrobial compounds, such as essential oils and plant-derived extracts, may represent a promising complementary strategy to conventional disinfectants, especially in the context of increasing antimicrobial resistance and environmental sustainability concerns.

Overall, a multimodal approach remains essential to effectively interrupt transmission pathways within healthcare settings and to ensure long-term improvement in infection control outcomes.

## Figures and Tables

**Table 1:** Positive microbiological samples and identified bacterial species recovered from high-touch critical surfaces in operating rooms.

Analyzed Surface	Positive Samples (n)	Isolated Microorganisms
Operating table	2	*Staphylococcus warneri* (1); *Staphylococcus epidermidis* (1)
Medication table	2	*Staphylococcus epidermidis* (2)
Ventilation grille	2	*Micrococcus luteus* (1); *Staphylococcus epidermidis* (1)
Multiparameter monitor	1	*Staphylococcus epidermidis* (1)
Surgical lamp	1	*Staphylococcus warneri* (1)

**Table 2:** Positive hand samples and identified bacterial species isolated from healthcare workers across different hospital facilities of the ASL of Caserta.

Hospital Facility	Positive Samples (n)	Isolated Microorganisms
Marcianise	35	*Staphylococcus epidermidis* (10), *Staphylococcus aureus* (4), *Staphylococcus haemolyticus* (4), *Staphylococcus warneri* (3), *Staphylococcus hominis* (3), *Staphylococcus pseudintermedius* (1), *Bacillus pumilus* (1), Enterobacteriaceae (10)
Piedimonte Matese	16	*Staphylococcus epidermidis* (7), *Staphylococcus haemolyticus* (1), *Staphylococcus hominis* (1), *Staphylococcus warneri* (1), *Staphylococcus aureus* (1), *Enterobacteriaceae* (5)
Sessa Aurunca	11	*Staphylococcus epidermidis* (7), *Staphylococcus haemolyticus* (1), *Staphylococcus aureus* (1), Enterobacteriaceae (2)
Maddaloni	8	*Staphylococcus epidermidis* (3), Enterobacteriaceae (5)

Samples were collected from healthcare personnel working in operating rooms, intensive care units, and internal medicine wards. Microorganisms were identified by MALDI-TOF mass spectrometry and include both Gram-positive and Gram-negative species. Enterobacteriaceae are reported as a family-level group.

**Table 3:** Blood culture positivity and microorganisms isolated across hospital facilities.

Hospital Facility	Positive Samples (n)	Isolated Microorganisms
Marcianise	171	*Staphylococcus* spp. (82), Gram-negative bacteria (33), Other microorganisms (56)
Piedimonte Matese	150	*Staphylococcus* spp. (53), Gram-negatives bacteria (27), Other microorganisms (70)
Sessa Aurunca	104	*Staphylococcus* spp. (48), Gram-negative bacteria (20), Other microorganisms (36)
Maddaloni	100	*Staphylococcus* spp. (56), Gram-negative bacteria (39), Other microorganisms (5)

**Table 4:** Quantitative evaluation of the in vitro antibacterial activity of HLE, GLE, GTE, PFE, and TEO compared to SPC against colonizing bacterial strains isolated from healthcare workers: *S. epidermidis* HSSe1, *S. hominis* HSSh1, *S. aureus* HSSa1, and *K. pneumoniae* HSKp1.

Microorganism		GTE [mg mL^−1^]	PFE [mg mL^−1^]	TEO [µL mL^−1^]	SPC [mg mL^−1^]
*S. epidermidis* HSSe1	MIC	2	10	0.25	2
	MBC	10	n.d.	1	5
*S. hominis* HSSh1	MIC	2	10	0.25	1
	MBC	10	>20	2.5	5
*S. aureus* HSSa1	MIC	1	5	0.25	2
	MBC	10	>20	1	5
*K. pneumoniae* HSKp1	MIC	20	20	2	0.25
	MBC	>20	>20	5	1

MIC, minimum inhibitory concentration; MBC, minimum bactericidal concentration; GTE, green tea extract; PFE, pomegranate extract; TEO, thyme essential oil; SPC, sodium percarbonate.

## Data Availability

The original contributions presented in this study are included in the article and Supplementary Material. Further inquiries can be directed to the corresponding authors.

## Supplementary Materials

The following supporting information can be downloaded at: https://www.mdpi.com/article/10.3390/microorganisms14061382/s1, Table S1. Percentage distribution of bacterial species isolated from healthcare workers’ hand samples across different hospital facilities of the ASL of Caserta. Table S2. Percentage distribution of bacterial species isolated from blood culture positivity samples across different hospital facilities of the ASL of Caserta. Table S3. Antimicrobial susceptibility against *Staphylococcus* spp. strains isolated from the hands of healthcare workers. Table S4. Antimicrobial susceptibility against *K. pneumoniae* isolated from the hands of healthcare workers.
