# Supplementary material for: Monitoring Hygiene Protocols and Exploring Alternatives to Counteract Resistant Pathogens: A Case Study from Southern Italy on Healthcare-Associated Infection Control
Source: Microorganisms. 2026 Jun 22;14(6):1382. doi: 10.3390/microorganisms14061382 (PMC13305197; doi:10.3390/microorganisms14061382)
Supplement: Supplementary file 1 [file microorganisms-14-01382-s001.zip › microorganisms-4353543-supplementary.pdf]

Supplementary Materials

Monitoring Hygiene Protocols and Exploring Alternatives to Counteract Resistant Pathogens: A Case Study from Southern Italy on Healthcare-Associated Infections Control

Table S1. Percentage distribution of bacterial species isolated from healthcare workers' hand samples across different hospital facilities of the ASL of Caserta.

Isolated microorganisms	Percentage (%)
Staphylococcus spp.	67
Klebsiella pneumoniae	17
Acinetobacter baumannii	9
Pseudomonas aeruginosa	4
Other microorganisms	3

Table S2. Percentage distribution of bacterial species isolated from blood culture positivity samples across different hospital facilities of the ASL of Caserta.

Isolated microorganisms	Percentage (%)
Staphylococcus epidermidis	16
Staphylococcus aureus	15
Staphylococcus hominis	9
Klebsiella pneumoniae	9
Pseudomonas aeruginosa	6
Acinetobacter baumannii	5
Staphylococcus haemolyticus	5
Other microorganisms	35

Table S3. Antimicrobial susceptibility against *Staphylococcus* spp. strains isolated from the hands of healthcare workers.

Antimicrobial agent	*S. aureus* HSSa1		*S. hominis* HSSh1		*S. epidermidis* HSSe1	
	**MIC**	**INT.**	**MIC**	**INT.**	**MIC**	**INT.**
Cefoxitin	POS	+				
Benzylpenicillin	> 0.25	R				
Oxacillin	> 2	R	≤ 0.25	S	> 2	R
Ceftaroline	0.5	S				
Gentamicin	≤ 0.5	S	≤ 0.5	S	≤ 0.5	S
Levofloxacin	> 4	R	≤ 0.12	I	0.25	I
Inducible clindamycin resistance	NEG	-	NEG	-	POS	+
Erythromycin	1	S	1	S	> 4	R
Clindamycin	≤ 0.12	S	0.25	S	0.25	R
Linezolid	2	S	4	S	2	S
Daptomycin	1	S	≤ 0.12	S	0.5	S
Teicoplanin	≤ 0.5	S				
Vancomycin	≤ 0.5	S	1	S	2	S
Tetracycline	≤ 1	S	≤ 1	S	> 8	R
Tigecycline	0.25	S	≤ 0.12	S	0.5	S
Fusidic acid	≤ 0.5	S	≤ 0.5	S	≤ 0.5	S
Mupirocin	≤ 1					
Rifampicin	≤ 0.03	S	≤ 0.03	S	≤ 0.03	S
Trimethoprim/ Sulfamethoxazole	≤ 10	S	≤ 10	S	≤ 10	S

MIC, minimum inhibitory concentration; INT., The interpretation of antimicrobial susceptibility testing was performed according to the criteria established by the European Committee on Antimicrobial Susceptibility Testing (EUCAST) as follows: S, susceptible; I, intermediate; R, resistant.

Table S4. Antimicrobial susceptibility against *K. pneumoniae* isolated from the hands of healthcare workers.

Antimicrobial agent	*K. pneumoniae* HSKp1	
	**MIC**	**INT.**
Extended-Spectrum Beta-Lactamase (ESBL)	POS	+
Temocillin	8	I
Ampicillin	> 16	R
Amoxicillin/Clavulanic acid	> 32	R
Ampicillin/Sulbactam	> 16	R
Piperacillin/Tazobactam	> 64	R
Cefuroxime	32	R
Cefuroxime axetil	32	R
Cefoxitin	> 32	R
Cefotaxime	≤ 0.25	S
Ceftazidime	4	S
Ceftriaxone	≤ 0.25	S
Ceftazidime/Avibactam	0.5	S
Ceftolozane/Tazobactam	1	S
Cefepime	1	S
Aztreonam	≤ 1	S
Ertapenem	≤ 0.12	S
Imipenem	0.5	S
Meropenem	≤ 0.25	S
Imipenem/Relebactam	0.5	S
Meropenem/Vaborbactam	≤ 0.5	S
Amikacin	4	S
Gentamicin	> 8	R
Tobramycin	> 8	R
Ciprofloxacin	> 2	R
Levofloxacin	> 4	R
Moxifloxacin	> 4	R
Colistin	> 4	R
Trimethoprim/Sulfamethoxazole	> 160	R

MIC, minimum inhibitory concentration; INT., The interpretation of antimicrobial susceptibility testing was performed according to the criteria established by the European Committee on Antimicrobial Susceptibility Testing (EUCAST) as follows: S, susceptible; I, intermediate; R, resistant.
